# Polytrauma scoring revisited: prognostic validity and usability in daily clinical practice

**DOI:** 10.1007/s00068-022-02035-5

**Published:** 2022-07-10

**Authors:** Robert Girshausen, Klemens Horst, Christian Herren, Felix Bläsius, Frank Hildebrand, Hagen Andruszkow

**Affiliations:** Department of Orthopedics, Trauma and Reconstructive Surgery and Harald Tscherne Laboratory, University Hospital RWTH Aachen, Pauwelsstraße 30, 52074 Aachen, Germany

**Keywords:** Severe trauma, Polytrauma, Trauma scoring, Clinical assessment, Trauma mortality

## Abstract

**Purpose:**

Scores are widely used for the assessment of injury severity and therapy guidance in severely injured patients. They differ vastly regarding complexity, applicability, and prognostic accuracy. The objective of this study was to compare well-established with more recently developed trauma scores as well as intensive care unit (ICU) scores.

**Methods:**

Retrospective analysis of severely injured patients treated at a level I trauma centre from 2010 to 2015. Inclusion criteria: Age ≥ 18 years, Injury Severity Score ≥ 16 and ICU treatment. Primary endpoint was in-hospital mortality. Several scores (ISS, APACHE II, RTS, Marshall Score, SOFA, NISS, RISC II, EAC and PTGS) were assessed to determine their predictive quality for mortality. Statistical analysis included correlation analysis and receiver operating characteristic (ROC).

**Results:**

444 patients were included. 71.8% were males, mean age was 51 ± 20.26 years. 97.4% sustained a blunt trauma. The area under the ROC curve (AUROC) revealed RISC II (0.92) as strongest predictor regarding mortality, followed by APACHE II (0.81), Marshall score (0.69), SOFA (0.70), RTS (0.66), NISS (0.62), PTGS (0.61) and EAC (0.60). ISS did not reach statistical significance.

**Conclusions:**

RISC II provided the strongest predictive capability for mortality. In comparison, more simple scores focusing on injury pattern (ISS, NISS), physiological abnormalities (RTS, EAC), or a combination of both (PTGS) only provided inferior mortality prediction. Established ICU scores like APACHE II, SOFA and Marshall score were proven to be helpful tools in severely injured trauma patients.

## Purpose

Treatment of severely injured patients remains one of the most challenging tasks in trauma care. Despite extensive research and various improvements, there is still disagreement on the optimal treatment algorithm [1]. In this respect, an early assessment of patients’ conditions seems crucial to avoid complications, to prioritise transportation, to guide initial surgical strategies, and ultimately to reduce mortality [2, 3]. Apart from guiding treatment strategies, there is a versatile applicability for scoring systems. When treatment resources are limited, scores can be useful as a triage tool to identify patients with the highest treatment priority [4]. Moreover, quality assessment and external validation of trauma care is only possible if trauma patterns and the associated condition of the patients can be objectified [4]. Furthermore, accurate scoring remains an important precondition for clinical trauma research.

Existing trauma scoring systems can be categorized as primarily anatomical, physiological or combined [4]. Another crucial aspect is complexity which differs vastly between the well-established scores [5]. While some scores can be applied in the early treatment phase, and even in the pre-hospital phase, others are only applicable once the diagnostic process has been finished [5]. A possible characterization of the perfect trauma score includes high validity, reliability, and prognostic accuracy while being based on widely available data. Moreover, it should be easily applicable and suitable for all injury patterns and age categories [6].

As a recent example of an attempt to simplify trauma scoring, the early appropriate care (EAC) protocol has been introduced. It is based on a simple score only requiring three parameters (pH, BE, lactate) from blood gas analysis at the time of hospital admission [7].

As early outcome assessment is beneficial for severe trauma, this study intended to compare several well-established as well as more recently developed scoring systems regarding their prognostic accuracy towards in-hospital mortality. We hypothesized that scores currently used in trauma care may not provide a sufficient mortality prediction. Furthermore, we hypothesized that higher complexity and a higher number of included parameters could lead to an improved prognostic accuracy.

## Methods

### Inclusion and exclusion criteria

A retrospective analysis of severely injured patients, defined as Injury Severity Score (ISS) ≥ 16 points, was performed. All patients were primarily admitted to a level I trauma centre between January 1, 2010 and December 31, 2015. Further inclusion criteria were admission to intensive care unit (ICU) within the first 24 h, treatment in our hospital for at least 48 h and age ≥ 18 years. Exclusion criteria were secondary admissions from other hospitals and trauma due to primarily medical or neurological conditions (i.e., myocardial infarction, ischemic or haemorrhagic stroke).

**Fig. 1:**
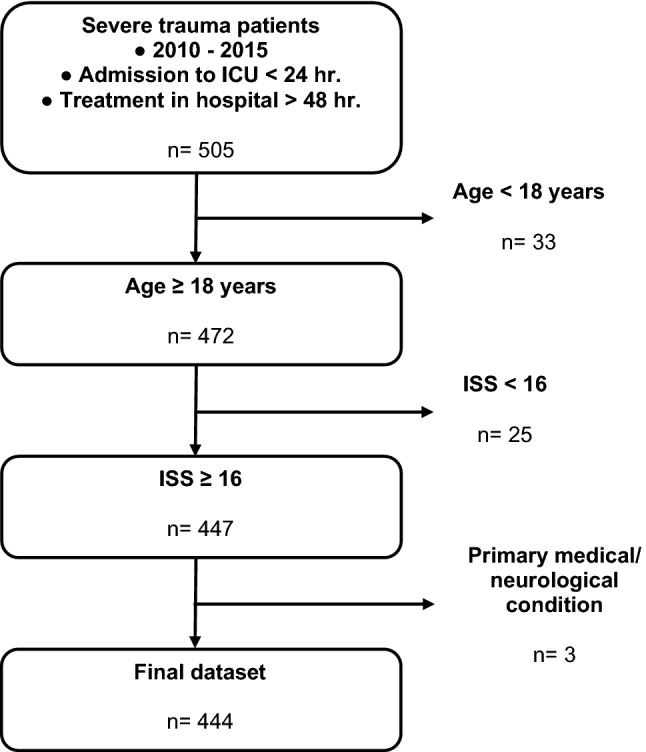
Inclusion and exclusion criteria

An overview of the inclusion and exclusion process is shown in Fig. 1.

### Data collection and evaluated scores

The data included demographics, detailed injury patterns based on clinical and radiographic findings, laboratory findings, pre-hospital and in-hospital management, surgical treatment, ICU treatment, and outcome at time of hospital discharge. Patients were treated according to current ATLS guidelines and total body CT scan was used routinely as part of our polytrauma protocol.

We evaluated the following scoring systems: Injury Severity Score (ISS), New Injury Severity Score (NISS), Acute Physiology and Chronic Health Evaluation II (APACHE II), Revised Trauma Score (RTS), Sequential Organ Failure Assessment (SOFA), Marshall score (Multiple Organ Dysfunction Score, MODS), Early Appropriate Care (EAC), Revised Injury Severity Classification II (RISC II) and PolyTrauma Grading Score (PTGS) in our analysis.

ISS and NISS were calculated based on the AIS 2005 version for the German Trauma Registry (TR-DGU) [8]. The AIS is an anatomically based scoring system which classifies injuries for different body regions by assigning a score on a six-point-scale: 1 (minor) to 6 (unsurvivable). To calculate the ISS, identification of the three most seriously injured body regions is necessary. The highest AIS score of each of these regions is squared and summed up to a total score with a maximum of 75 [9, 10]. NISS has been introduced as a modification of ISS. In contrast to ISS, NISS is calculated using the three highest AIS scores regardless of the body region [11]. APACHE II is calculated using 12 parameters (body temperature, mean arterial blood pressure, heart rate, respiratory rate, oxygenation, arterial pH, serum sodium, serum potassium, serum creatinine, haematocrit, white blood count and GCS) as well as age and previous health status [12]. The SOFA score includes six organ systems: Respiratory (Horowitz index), circulatory (blood pressure or need for vasopressors/ inotropes), hepatic (serum bilirubin), renal (serum creatinine), coagulation (platelet count) and central nervous system (Glasgow Coma Scale, GCS). Each organ system is assigned a maximum of 4 points which are added to a total score. Multi-organ failure (MOF) is usually defined as a SOFA score ≥ 3 points for a given organ system and at least two organ systems failing at the same time [13]. The Marshall score (MODS) includes the same organ systems as SOFA. Unlike SOFA, the Marshall score uses pressure adjusted heart rate (compound of heart rate, central venous pressure and mean arterial pressure) for the circulatory organ system [14, 15]. Similarly to SOFA, MOF is defined as a score ≥ 3 points in at least two organ systems [14]. RTS consists of the physiological parameters GCS, systolic blood pressure and respiratory rate. EAC is based on pH, BE and lactate. Thereby, patients are classified as low or high risk towards the development of in-hospital complications determining the surgical treatment strategy [16]. RISC II was developed to compare the injury severity of patients with different injury patterns. RISC II includes anatomical (AIS of worst injury/ second worst injury/ head injury), physiological (blood pressure, motor function, pupil size/ reactivity, INR, base deficit, haemoglobin) and other parameters (age, sex, ASA score, trauma mechanism, necessity of CPR) [17]. PTGS assesses injury severity using a combination of six anatomical or physiological parameters (systolic blood pressure, INR, base deficit, platelet count, number of administered PRBC units and NISS) [2]. An overview of all investigated scoring systems is presented in Table 1.

**Table 1:** Overview of investigated scoring systems

Score	Score category	Number of required parameters*	Availability**	Primary publication
ISS	Anatomical	6	Trauma Room	[9]
APACHE II	Physiological	14	ICU	[12]
RTS	Physiological	3	Pre Hospital	[26]
Marshall Score	Physiological	7	ICU	[14]
SOFA Score	Physiological	7	ICU	[13]
NISS	Anatomical	6	Trauma Room	[11]
RISC II	Combined	19	Trauma Room	[19]
EAC	Physiological	3	Trauma Room	[16]
PTGS	Combined	11	ICU	[2]

*Includes all parameters that are necessary for calculating the score (i.e., ISS is a compound of all six AIS regions, therefore six parameters are required)

**First point in time, at which the score can be calculated. Preconditions are the possibility of CT imaging and point of care laboratory measurements (blood gas analysis, INR) in the emergency department

### Endpoint

Mortality was assessed for all patients that deceased during the clinical course.

### Statistical analysis

Incidences were presented as counts and percentages while continuous variables were presented as mean and standard deviation (SD). The predictive accuracy of scores and other variables was assessed using receiver operating characteristic (ROC) curves and the area under the ROC curve (AUROC). A two-sided p-value < 0.05 was considered statistically significant. All calculations were performed with SPSS statistical software package (version 25, IBM Inc., Armonk, NY, USA).

## Results

### Demographics

444 patients were included in the present study. 71.8% were male. Mean age was 51 ± 20.26 years. 97.4% sustained blunt trauma. Main reasons for the trauma were traffic accidents as well as falls from great height (Table 2).

**Table 2:** Descriptive data

Number of Cases	444
Age (years)	51 ± 20.26
Male gender	319 (71.8%)
Blunt Trauma Mechanism	410 (97.4%)
Type of Injury	
-Traffic: Car	74 (16.7%)
-Traffic: Motorbike	55 (12.4%)
-Traffic: Bicycle	31 (7.0%)
-Traffic: Pedestrian	40 (9%)
-Low Fall (< 3 m)	98 (22.1%)
-High Fall (> 3 m)	74 (16.7%)
-Burns/ Explosion	12 (2.7%)
ISS	25 ± 8.43
NISS	28.9 ± 9.71
mAIS	3.95 ± 0.81
AIS Head/ C-Spine ≥ 3	252 (56.9%)
Length of stay in hospital (days)	25.58 ± 25.67
Length of stay in ICU (days)	16.1 ± 22.6
Acute Respiratory Distress Syndrome (ARDS)	36 (8.1%)
Multi Organ Failure (MOF)	102 (23%)
Hospital Mortality	116 (26.1%)
Cause of Death	
-Fatal Cerebral Injury	62 (62.6%)
-Cardiocirculatory Failure	18 (18.2%)
-Multi Organ Failure	11 (11.1%)
-Unsuccessful CPR/ Termination of Treatment	8 (8.1%)

Continuous variables given as mean ± standard deviation, categorical variables as number of cases and percentage. Due to incomplete data the total number of cases for calculation of percentages can differ from 444

*ISS* injury severity score, *NISS* new injury severity score, *mAIS* maximum abbreviated injury scale, *AIS* abbreviated injury scale, *ICU* intensive care unit, *CPR* cardiopulmonary resuscitation

### Injury severity

The ISS was 25 ± 8.43 points, whereas NISS was 28.9 ± 9.71 points. Regarding the AIS, head and neck were the most severely injured regions (2.66 ± 1.92), followed by chest (1.78 ± 1.62), extremities (1.51 ± 1.48), abdomen (0.74 ± 1.29), face (0.57 ± 1.05) and soft tissue (0.44 ± 0.92).

### ARDS and MOF

36 patients (8.1%) developed ARDS according to the Berlin definition [18] during the clinical course, most frequently diagnosed on the second day after admission. 102 patients (23%) developed MOF according to the SOFA score.

### Mortality

116 patients (26.1%) died during the clinical course. 35.3% of deaths occurred within the first 24 h after admission. Deceased patients showed a significantly increased ISS compared to the survivors (28.37 ± 10.82 vs. 23.81 ± 7.04; *p* < 0.001). The most common causes of death were fatal cerebral injuries, followed by cardiocirculatory failure, MOF, unsuccessful CPR, or termination of treatment (Table 2).

### Predictive quality of trauma scores

**Fig. 2:**
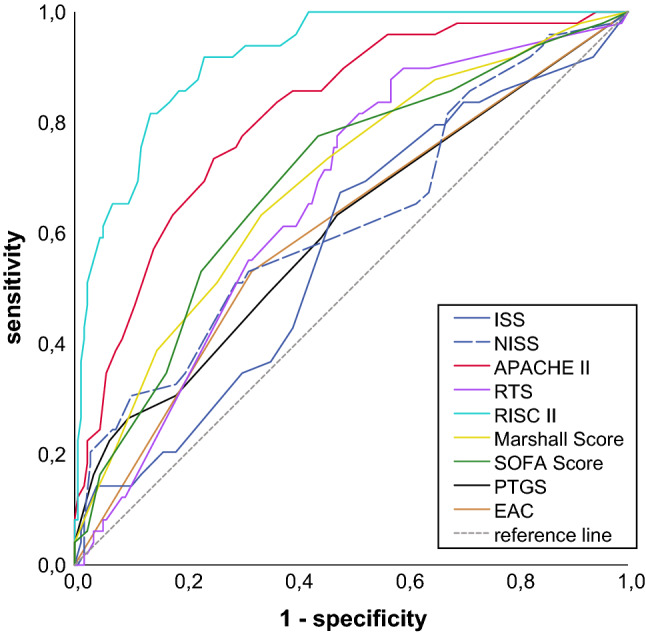
ROC curves of scoring systems towards mortality. ROC curves for RISC II, APACHE II, Marshall Score, SOFA, RTS, NISS, PTGS and EAC regarding mortality

All scores showed a significant correlation with mortality as shown in Table 3. To investigate the prognostic accuracy towards mortality we used the receiver operating characteristic (ROC) method. The ROC curves are shown in Fig. 2. The calculated AUROC values are shown in Table 4.

**Table 3:** Correlation of scoring systems towards mortality

Scoring System	Correlation coefficient	*p* value
ISS	0.21	< 0.001
APACHE II	0.45	< 0.001
RTS	0.25	< 0.001
Marshall Score	0.24	< 0.001
SOFA	0.27	< 0.001
NISS	0.25	< 0.001
RISC II	0.66	< 0.001
EAC	0.20	< 0.001
PTGS	0.22	< 0.001

Correlation coefficient and p-value for RISC II, APACHE II, Marshall Score, SOFA, RTS, NISS, PTGS, EAC and ISS. Spearman's rank correlation coefficient was used

**Table 4:** Area under the ROC curve (AUROC)

Scoring system	AUROC	*p* value	95%-CI
RISC II	0.92	< 0.001	0.88–0.96
APACHE II	0.81	< 0.001	0.75–0.88
Marshall Score	0.69	< 0.001	0.61–0.78
SOFA Score	0.70	< 0.001	0.61–0.78
RTS	0.66	0.001	0.58–0.74
NISS	0.62	0.009	0.53–0.72
PTGS	0.61	0.022	0.51–0.70
EAC	0.60	0.024	0.51–0.7
ISS	0.57	0.118	0.48–0.66

AUROC (area under the ROC curve), *p* value and 95% confidence interval for RISC II, APACHE II, Marshall Score, SOFA Score, RTS, NISS, PTGS, EAC and ISS

AUROC revealed RISC II as strongest predictor regarding mortality, followed by APACHE II, Marshall score, SOFA, RTS, NISS, PTGS and EAC.

## Discussion

Even though all investigated scores revealed a significant correlation with mortality, their predictive accuracy in the ROC analysis differed vastly. RISC II provided the strongest predictive capability towards mortality. In comparison, more simple scores focusing on injury pattern (ISS, NISS), physiological abnormalities (RTS, EAC), or a combination of both (PTGS) only provided a less precise mortality prediction.

RISC II showed the highest AUROC for mortality. It was originally developed for risk adjustment in the German trauma registry and was updated in 2014 [17, 19]. Requiring 19 parameters and using multiple weighting constants, the RISC II represents the most complex of the investigated scores. Nonetheless, it is possible to gather all necessary data in the trauma room phase. After initial validation using a large German trauma patient cohort and showing a high predictive accuracy comparable to our data [17], this result was also confirmed in a Spanish retrospective analysis [20].

The established ICU scores APACHE II, Marshall, and SOFA score all show a relatively high AUROC indicating that they may also be useful for mortality prediction in trauma patients. While APACHE II was designed to predict mortality in ICU patients, the Marshall and SOFA scores focus on the detection of MOF [12, 21]. A clear advantage of these scores is that they are well-established in many ICUs and are often routinely used to monitor the course of the patients’ physiological state. A disadvantage is their dependence on laboratory parameters, which usually does not allow their use in the early trauma room phase.

The SOFA score is routinely used to monitor MOF in the ICU [22, 23]. The utility of SOFA and Marshall score for outcome prediction in a mixed ICU population was demonstrated in several prospective studies [15, 21]. Looking specifically at trauma patients, SOFA was found to be helpful in identifying patients with high risk of prolonged ICU stay or death, especially in the first four days after admission [24]. While our study focused on prediction of in-hospital mortality and did not include secondary outcomes such as MOF or ICU length of stay, these could be interesting targets for further research.

Similar to our findings, in a retrospective analysis of trauma patients in rural India APACHE II showed a higher AUROC and therefore better prediction of mortality compared to RTS and TRISS. The predictive capability was even better on day one after admission than at the time of admission [3]. This reveals a dilemma: If a score is obtained later in the clinical course, the manifestation of complications and therefore the predictive quality rises, while early prediction is crucial to clinical decision making. The study further emphasises the possible usefulness of ICU scores in polytrauma patients. A retrospective study found a better predictive capability of APACHE II towards severe SIRS and sepsis in severe trauma patients compared to ISS, NISS and prothrombin time as a marker of coagulopathy [25]. This is, however, not surprising since all SIRS criteria are part of the APACHE II score [12].

In our study, the simple physiological scores RTS and EAC showed a much lower AUROC towards mortality than the aforementioned scores. RTS was designed to be used for simple outcome evaluation in trauma patients. It is the only score that can be applied in a pre-hospital as well as in an in-hospital setting. As another advantage it only requires three vital signs. Therefore, it was proposed as a pre-hospital triage tool [4, 6, 26]. When compared to other simple trauma scores designed for pre-hospital use such as the New Trauma Score, using only slightly different parameters, RTS provided similar results [27]. In a Korean prospective cohort study RTS showed a relatively strong predictive capacity towards mortality which we could not confirm in our patient collective [27].

The EAC protocol was developed to select stable patients for early definitive stabilization and unstable patients who first require further resuscitation based on acidosis [16]. The score is a composite of pH, base excess and lactate which are closely connected to each other. Therefore, the superiority of the EAC score over the individual parameters can be questioned. In our patient collective, EAC was inferior to all other scores predicting mortality except ISS. Still, it must be noted that EAC was not designed and validated for this purpose.

Overall, RTS and EAC represent a multitude of relatively simple trauma scores that have been developed in the past decades. Despite several limitations they can be helpful, especially in the early phase of trauma care and as a triage tool. Nevertheless, efforts to develop very simple trauma scores by combining few physiological or laboratory parameters have not yet led to convincing results and will most likely not be successful in the future due to the complexity and individuality of trauma patients.

PTGS was designed specifically for the assessment of polytrauma patients to guide their treatment. It is easily applicable once laboratory tests are available, and has defined cut-offs for stable, borderline, and unstable patients [2]. Nevertheless, in our patient collective PTGS could not provide a sufficient prediction of mortality in comparison to more complex scores. However, PTGS was not designed and validated for this purpose either.

The ISS is the most commonly used score for grading trauma severity worldwide [5]. Itis merely based on injury patterns [9]. The NISS is a slight modification to include more than one severe injury in one anatomical region [11]. In a review article focusing on scores for quality assessment, the broad acceptance of the ISS is seen as an obstacle to establishing more precise scores in trauma care [5]. The varying influence on mortality of certain injuries with the same AIS value and the inhomogeneous mortality rates associated with similar ISS scores are criticised [4]. Even though NISS was shown to be more precise in mortality prediction it never replaced ISS in clinical and scientific practise [28]. Both scores showed a low predictive accuracy in our patient cohort, ISS not even being significant in the ROC analysis. A possible explanation is the disregard of physiological changes in ISS and NISS.

Regarding a review of different trauma scores, the use of scores using purely anatomical injury patterns is generally questioned [4]. Potential problems may be an under- or overestimation of trauma severity due to erroneous injury classification, dependence of the injury-associated mortality on the quality of care in a certain EMS or hospital system, and the disregard of the patient’s individual response reflected by physiological parameters. Therefore, the authors advocate for the use of trauma scores that include anatomical as well as physiological parameters [4].

### Interobserver variability

Due to differences in complexity and objectifiability of the underlying parameters, varying interobserver variability between the investigated scores must be assumed. Scores based on laboratory parameters or vital signs are less prone to high interobserver variability than scores based on injury patterns since the latter require clinical or radiological detection as well as classification. A study evaluating 54 trauma course participants (mostly orthopedic trauma and general surgeons) showed high interobserver variability in obtaining AIS, ISS and NISS based on trauma cases. Interobserver variability differed depending on the speciality of the participant and injury regions [29].

### Comparison of patient collectives

A comparison of the patient collective in our study and 39,346 German trauma patients in the German Trauma Registry showed that gender (71.8% vs. 71% male patients), trauma mechanism (97.4% vs. 96% blunt trauma) and age (51 vs. 49.2 years) were very similar [30]. We therefore believe that our results are well applicable to other institutions with similar patient populations and treatment protocols while applicability in vastly different systems of trauma care might be limited.

A noticeable difference was observed in terms of 30-days mortality (25.2% vs. 11.4%). One explanation is the higher injury severity (ISS 25 vs. 19.7) in our patient collective. Accordingly, all patients had an ISS ≥ 16 while only 59% of patients in the German Trauma Registry fulfilled this criterion [30].The prevalence of severe head injury (AIS head/c-spine ≥ 3) was higher in our patient collective (56.9 vs. 39%). Severe cerebral injury was found to be the most common cause of death. Furthermore, data from the German Trauma Registry show that level I trauma centres have a higher overall mortality than hospitals with lower level of care [30]. This is most likely due to patient selection and secondary admissions to a higher level of care. Furthermore, in contrast to many trauma studies we did not exclude early mortalities.

### Limitations

Due to the retrospective design of this study, only clinical routine data could be included in the analysis. While epidemiologic data, radiographic- and laboratory findings were relatively complete, vital signs and more complex data like the Glasgow Coma Scale were missing more frequently. Similar problems are described for the German Trauma Registry (Traumaregister der Deutschen Gesellschaft für Unfallchirurgie, TR-DGU). Between 2006 and 2014 an average of 12.2% of data were missing [30]. To improve documentation quality, standardized protocols and digital documentation tools can be useful in fast-moving and often stressful environments like a trauma room or operation theatre.

Our local trauma registry which was the basis for this study includes trauma patients in the years 2010–2015. To identify potential changes in trauma populations over time, we compared annual reports of the German trauma registry from 2013 [31] and 2021 [32]: Mean age increased from 47,6 to 54.2 years while the proportion of male patients and blunt trauma did not change. Mean ISS and hospital mortality only slightly increased from 17.0 to 18.4 and 10.0% to 12.7%, respectively. Prehospital infusion volume slightly decreased from 698 to 608 ml. In 2020, the use of tranexamic acid increased in comparison to the previous years. Overall, we believe that these changes do not limit the applicability of our results to current trauma patients.

A limitation of most studies investigating trauma scoring including this study is their focus on short term outcomes like mortality, MOF or ARDS. Therefore, little is known about the prediction of long term outcomes of patients surviving the acute phase [4]. Future research is necessary to investigate the prediction of outcomes such as the ability to return to work or the functional outcome after rehabilitation.

## Conclusions

A standardized approach to the treatment of severe trauma is needed to further reduce morbidity and mortality in this patient population. A key precondition remains the precise distinction between stable and unstable patients to prevent adverse outcomes, especially to reduce mortality. Established and widely used trauma scores, especially the ISS, lack in predictive accuracy towards mortality. Therefore, the role of the ISS as the “gold standard” in trauma scoring should be challenged. High prognostic accuracy as provided by the RISC II score involves a high grade of complexity. Therefore, its use in daily clinical practise is limited. More recently developed, simple scores provide a low prognostic accuracy. Nevertheless, their potential use as triage and early prognostication tools with the use of more complex scores later on should be discussed. ICU scores, which are routinely used in many hospitals, also have a relatively high prognostic accuracy for mortality in trauma patients. This makes them a promising subject for further trauma scoring research. Despite extensive research in the field of trauma scoring, the ideal score is yet to be developed. The validation process for future trauma scores should include prospective studies that can reveal an improved outcome for severely injured patients with the use of scores and standardised treatment protocols.
